# Carbon-Negative Construction Material Based on Rice Production Residues

**DOI:** 10.3390/ma18153534

**Published:** 2025-07-28

**Authors:** Jüri Liiv, Catherine Rwamba Githuku, Marclus Mwai, Hugo Mändar, Peeter Ritslaid, Merrit Shanskiy, Ergo Rikmann

**Affiliations:** 1 Cellula Ltd., Raua 1, 10124 Tallinn, Estonia; jyri.liiv@cellula.ee; 2 Department of Civil Engineering, Kenyatta University, Nairobi P.O. Box 43844-00100, Kenya; githuku.catherine@ku.ac.ke (C.R.G.); mmarclus@yahoo.com (M.M.); 3 Institute of Physics, University of Tartu, W. Ostwaldi 1, 50411 Tartu, Estonia; hugo.mandar@ut.ee (H.M.); peeter.ritslaid@ut.ee (P.R.); 4 Institute of Agricultural and Environmental Sciences, Estonian University of Life Sciences, Kreutzwaldi 5, 51014 Tartu, Estonia; merrit.shanskiy@emu.ee; 5 Institute of Chemistry, University of Tartu, Ravila 14A, 50411 Tartu, Estonia

**Keywords:** carbon-negative, construction material, rice husk ash, wood ash, compressive strength

## Abstract

This study presents a cost-effective, carbon-negative construction material for affordable housing, developed entirely from locally available agricultural wastes: rice husk ash, wood ash, and rice straw—materials often problematic to dispose of in many African regions. Rice husk ash provides high amorphous silica, acting as a strong pozzolanic agent. Wood ash contributes calcium oxide and alkalis to serve as a reactive binder, while rice straw functions as a lightweight organic filler, enhancing thermal insulation and indoor climate comfort. These materials undergo natural pozzolanic reactions with water, eliminating the need for Portland cement—a major global source of anthropogenic CO_2_ emissions (~900 kg CO_2_/ton cement). This process is inherently carbon-negative, not only avoiding emissions from cement production but also capturing atmospheric CO_2_ during lime carbonation in the hardening phase. Field trials in Kenya confirmed the composite’s sufficient structural strength for low-cost housing, with added benefits including termite resistance and suitability for unskilled laborers. In a collaboration between the University of Tartu and Kenyatta University, a semi-automatic mixing and casting system was developed, enabling fast, low-labor construction of full-scale houses. This innovation aligns with Kenya’s Big Four development agenda and supports sustainable rural development, post-disaster reconstruction, and climate mitigation through scalable, eco-friendly building solutions.

## 1. Introduction

### 1.1. Environmental Footprint of Construction Sector

Annually, about 7 billion cubic meters of concrete are used worldwide. This makes concrete the most widely used construction material and the second most consumed material by humankind after water [1]. The construction sector and its supporting activities are major sources of greenhouse gases (GHG) [2,3]. They are responsible for at least one-third of total GHG emissions. These activities also account for about 25% of water used in concrete production processes. Examples include concrete mixing, equipment cleaning, and curing. The sector contributes to about 25% of solid waste generation [3]. Therefore, reducing GHG emissions from the construction industry is a key goal of the EU Climate Program for developing countries. The current work was carried out under the EU program “Climate policy objectives for developing countries” [4,5,6].

Concrete buildings often face indoor climate challenges. In high-altitude tropical climates with significant daily temperature changes, bricks are considered better than concrete. Bricks help maintain a more uniform indoor temperature throughout the day [7]. In low-altitude wet tropical climates with high humidity, concrete building envelopes tend to heat up under intense solar radiation. This effect is especially noticeable in buildings with concrete slab roofs. As a result, air conditioning energy use increases significantly [8].

### 1.2. The National Housing Policy of Kenya

Kenya’s population is growing rapidly. This growth puts pressure on an already strained housing system. Article 43 (1b) of the Constitution of Kenya [9] states that every person has the right to accessible and adequate housing. It also guarantees reasonable standards of sanitation. Decent, affordable, and adequate housing is recognized as a human right and a key part of the right to an adequate standard of living. According to Sessional Paper No. 3 of 2016 on the National Housing Policy for Kenya [10], adequate housing includes shelter with enough privacy, space, accessibility, and structural stability. It should also provide good environmental quality and health conditions. Housing must be located near work and basic services and be affordable—costing no more than 30% of monthly household income. Meeting this standard for all Kenyans is a huge task. It cannot be left entirely to the public or private sectors.

The Government’s Big Four Agenda offers a major opportunity. One of its goals is to support the annual construction of at least 500,000 affordable housing units [10]. This highlights the need for government and other stakeholders to support innovation, including research into using waste-derived materials as building products. Such efforts can contribute to the national housing target.

This project explores the potential of rice straw, rice husk ash, and wood ash—considered waste products—for constructing affordable houses. Using these materials can also help manage agricultural waste. Benefits to the community include access to good, low-cost housing. It also promotes skill development in building techniques and supports climate change mitigation by reducing reliance on conventional cement. Cement production releases large amounts of carbon dioxide (~900 kg CO_2_/ton of cement). Since our material uses ash that has already been generated, there are no additional GHG emissions associated with it, but carbon dioxide is absorbed from the surrounding atmospheric air as the material hardens.

### 1.3. Construction Materials with Organic Filler

High cement prices, along with environmental and indoor climate concerns, have increased interest in using local and waste-based materials for construction in developing countries. Sustainable housing in such regions means using materials that are low cost, renewable, and locally available. Although concrete remains the most common building material, earthen construction accounts for nearly 30% of housing in developing countries. This is due to its availability, low cost, and long-standing use [11].

The mechanical properties of earth-based materials, such as clayey soil, or dredging waste, can be improved. Adding low-cost, waste-based calcareous materials like blast furnace slag, or pozzolanic additives like treated pulverized glass, helps enhance performance [12]. Lateritic soil forms in tropical areas through the weathering of bedrock under heavy rainfall. It contains iron and aluminum oxides, with smaller amounts of manganese and titanium oxides. Laterite-based building blocks and bricks have been used in traditional African architecture for centuries [13]. By combining modern and traditional building methods, rural communities can benefit from affordable housing. This approach improves sustainability and sanitation [14].

The use of straw in building blocks dates back to the Roman Empire, particularly in arid parts of North Africa. Evidence shows that it has also been used in many other regions [15]. Studies in Burkina Faso show that houses built with clay–straw mixtures can save up to 8% annually on air conditioning energy compared to houses made of pure clay. This saving can increase further when roof insulation and other measures are applied [16]. However, mud and straw bricks are mostly produced manually. This makes labor costs high and the process time-consuming. Rice straw is a cheap and widely available material across the tropical region; its high silica content makes the material more resistant to termites. Besides straw, other organic fillers may be used depending on regional availability. For example, peat could be an option in some areas. In Africa, large peat resources exist in the Congo River basin. This region hosts the world’s largest tropical peatland [17]. Another widely available potential organic filler material is sugarcane bagasse.

### 1.4. Natural Sources of Silica and Lime

Rice straw is a promising material for making organic–mineral composite building blocks in tropical regions. Worldwide, about 500 million tons of rice straw are produced each year. Managing this waste in a sustainable and economically viable way is a challenge. Often, it is burnt in fields, which causes serious air pollution. Rice straw contains high levels of silica (68–83 wt% SiO_2_ in ash). Because of this, it decomposes slowly and is not suitable for composting. It also has low nutritional value for cattle and is poorly digestible [18].

Agro-waste-based gypsum hollow-core blocks made with rice straw have shown excellent resistance to termites and fungal attacks. These blocks also offer good acoustic, fire-resistant, and thermal insulation properties [19]. A study in Brazil tested particle boards made from wood (*Eucalyptus grandis*), bamboo (*Bambusa vulgaris*), and rice husk (*Oryza sativa*) with 8 wt% urea-formaldehyde adhesives. The rice husk samples showed the highest resistance to termite attacks (*Nasutitermes corniger*) and decay caused by brown-rot (*Gloeophyllum trabeum*) and white-rot fungi (*Trametes versicolor*). This resistance is likely due to the high carbon content (high C/N ratio) and inorganic components like silica in rice residues. These make the material hard to digest for termites [20].

Chemically, rice husk ash is similar to fumed silica. About 200 kg of rice husk is produced per metric ton of rice. Rice husk ash has a high ash content—up to 20 wt%. It contains 92–95 wt% silica. It is highly porous, lightweight, and has a very large specific surface area [21]. These features make it an excellent pozzolanic agent and additive for cement and concrete. It can also generate certified emission reductions (carbon credits). Although rice husk is used in the cement industry, much of it is still disposed of as waste. Farmers often burn it in open fields without any useful application. Finding productive uses for rice production residues is essential to improving the environmental impact of this economic sector globally. If transportation costs are acceptable, other waste materials can also be used. These include lime, calcareous ashes, pozzolanic fly and bottom ashes, spent zeolite, bentonite-based sorbents, and blast furnace slag—as long as they are free from toxic contaminants that could limit their use.

In this paper, we present a concept for an environmentally friendly, carbon-negative, and low-cost building material. It is based on locally available raw materials, mainly agricultural wastes. The material is developed for use in rural Africa. It supports passive cooling and stable indoor temperatures. It provides a comfortable and healthy indoor climate. The material is resistant to termites, increases construction productivity, and improves hygiene.

## 2. Materials and Methods

### 2.1. Materials

Calcareous ash [22,23], such as wood ash, is used as a binder in the material. Wood ash also contains significant amounts of alkali metal oxides (especially potassium), which create a strongly alkaline environment, thereby promoting pozzolanic reactions. As a pozzolanic agent, rice husk ash is the most suitable. It has high reactivity due to its very high SiO_2_ content and large specific surface area. Alternatively, rice straw ash and sugarcane bagasse ash can be used as pozzolans, as they also contain large amounts of SiO_2_ [24]. Chopped rice straw or sugarcane bagasse can be used as organic filler. Optionally, clayey lateritic soil can be added as additional filler (depending on its humic matter content).

The material uses wastes, and, optionally, easily available natural materials like lateritic soil. No Portland cement or any other carbon-positive binder is needed. The material is analogous with peat composites developed at the University of Tartu [25,26], based on calcareous kukersite oil shale ash as a binder and peat as organic filler; fumed silica was added to the ash in order to compensate for the deficiency in the oxides’ pozzolanic activity.

The combined effect of nano- and microscale silica and alkali metal oxides in the ash accelerate the setting and hardening processes of the material. When the mortar is mixed, the oxides of alkali metals dissolve, creating pH > 13. Then typical pozzolanic reactions occur, as in the case of Portland cement. The principal pozzolanic reactions taking place during the concrete hardening are the following (Equations (1)–(3)) [25,26]:

C_3_S + (1.3 + x)H → C_1.7_SH_x_ + 1.3CH (1)

C_2_S + (0.3 + x)H → C_1.7_SH_x_ + 0.3C (2)

F + A + 4CH → C_4_ (3)

After the formation of crystalline phases, the residual free lime in the mortar reacts with atmospheric CO_2_, giving CaCO_3_. This process results in the carbon negativity of the material.

The wood ash was obtained from one of the fruit processing firms in Kajiado County, Kenya, where the wood is used in the boilers. The rice straw was obtained from one of the largest Rice Irrigation Schemes in Kenya (Mwea Irrigation Scheme in Kirinyaga County, Wamumu, Central Kenya). Its moisture content was 25% and its density was 15 kg/m^3^. Before use, the rice straw was shredded to a length of 3–5 cm using the straw cutter present in the mixing apparatus. The rice husk ash was also obtained from the Mwea Rice Irrigation Scheme. It contained close to 90% SiO_2_.

### 2.2. Analyses of the Raw Materials

The X-ray diffraction and X-ray fluorescence (XRD-XRF) analyses of the raw materials were performed in the National Phytotherapeutics Research Centre of Kenyatta University, Nairobi, Kenya.

The XRF machine S1 TITAN (Bruker, Karlsruhe, Germany) was configured to the following conditions: 40 kV, 10 mA.

Elemental analysis of the rice husk ash at the Institute of Physics, University of Tartu, Tartu, Estonia, was performed by X-ray wavelength dispersive fluorescence spectroscopy (XRF) using a Primus ZSX-400 spectrometer (Rigaku, Tokyo, Japan). The phase composition of the ash was analyzed using X-ray diffraction (XRD), implementing Bragg–Brentano optical geometry and rotating Cu anode working at 8.1 kW on a SmartLab diffractometer (Rigaku, Tokyo, Japan).

### 2.3. Construction of the Test House

Due to the considerations shown above, we have decided in favor of the option of traditional formwork casting.

**Figure 1:**
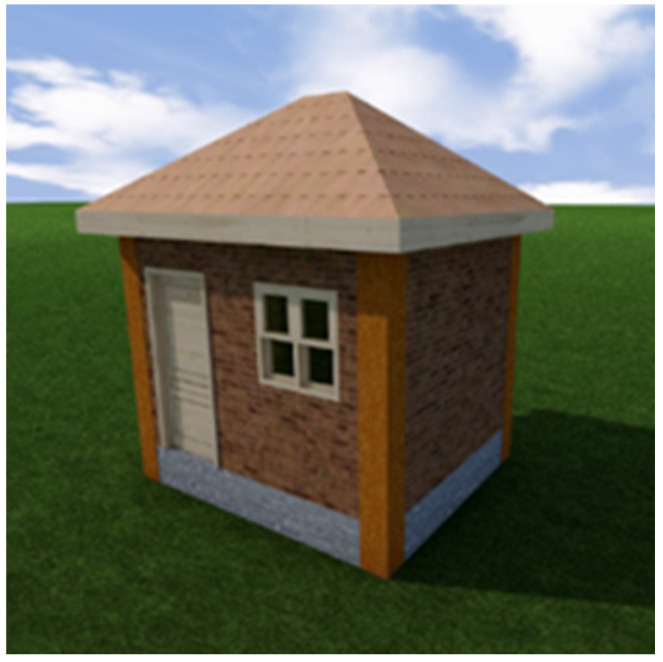
The model of the house that is being constructed.

Ordinary concrete tape or slab foundation is poured for the house. A reusable formwork is placed on it and filled with the composite, so all of the walls of the house are formed at once. After solidification, the formwork is removed and used in the construction of the next house. The walls are covered with a standard roof and equipped with windows, doors, and communications (see Figure 1).

A major challenge is to prepare a sufficient amount of the mixture. There may not be concrete plants in the destination area, or the may not be interested in producing a small amount of special mix. With a conventional mixer, making such a house is very time consuming and requires a lot of manual labor. Therefore, we decided to design a semi-automatic machine that crushes the straw, mixes the required amount of dry matter and water, prepares the mixture, and pumps it along the pipe into the formwork.

### 2.4. Equipment for the Mixing and Distribution of Composite Material

**Figure 2:**
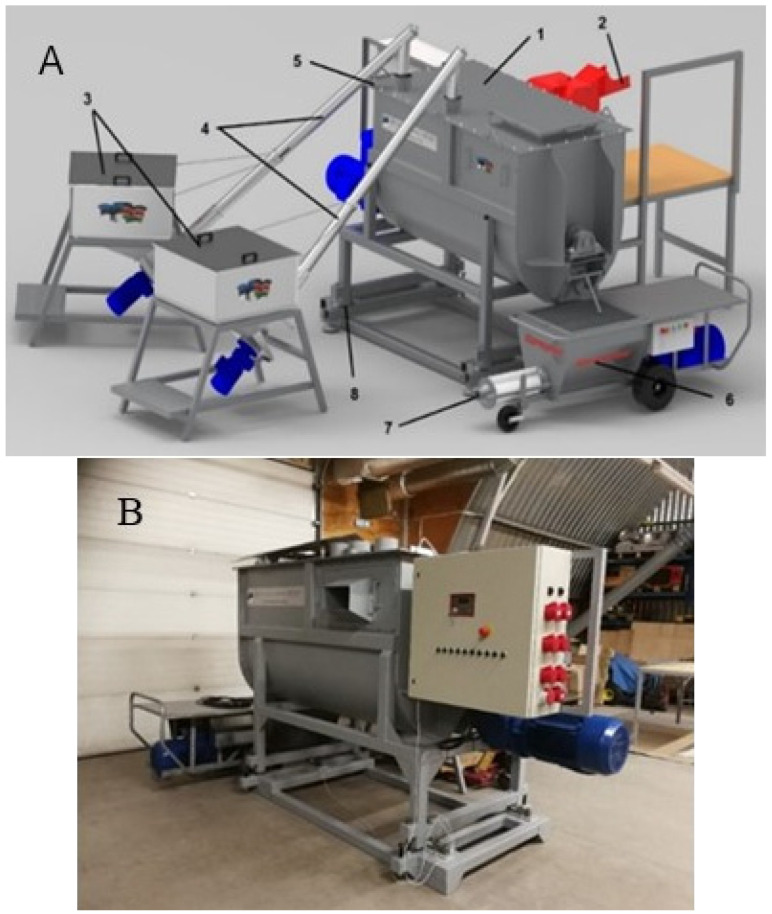
(**A**): schematic drawing of the mixing complex. 1—mixer, 2—straw cutter, 3—containers for dry ingredients, 4—screw conveyors, 5—water inlet, 6—concrete pump, 7—mixture outlet, 8—weighting system; (**B**): completed main mixer.

The apparatus designed during the project (Figure 2) is intended for the semi-automatic production and pumping of lightweight concrete with organic filler. The test machine allows a 100 m^2^ house to be erected in a few days using two workers. The machine was based on a medium-sized horizontal shaft mixer, in addition to which all the necessary units were constructed:

- screw conveyors for loading different components into the mixer
- pump for pumping the mixture
- rack for the mixer with weight sensors.

The original was manufactured by the French company Pellot (Turku, Finland).

The premade rice straw mixer was installed to cut the straw. The amount of components in the mixture added to the mixer is measured with the weight sensors under the mixer. The weight sensor control unit and the display are located on the control panel (Figure 2).

The technical parameters of the equipment include:

1. Horizontal shaft mixer:
  - power 15 kW, 3 phase 400 V
  - mixer shaft speed 16 rpm
  - full volume of the mixer container 1.5 m^3^, amount of mixture 0.7–1.0 m^3^.
2. Screw conveyor:
  - power 1.5 kW, 3 phase 400 V
  - auger speed 107 rpm
  - conveyor container capacity 300 L
  - productivity 24 L/min.
3. Mixture pump:
  - power 11 kW, 3 phase 400 V
  - container volume 175 L
  - pump capacity 120 L/min (measured with water)
  - hose ID 65 mm, length 15 m.
4. Straw crusher:
  - power 4 kW, 3 phase 400 V
  - rotation speed 2870 rpm.

## 3. Results

### 3.1. Elemental Analysis of the Raw Materials

**Figure 3:**
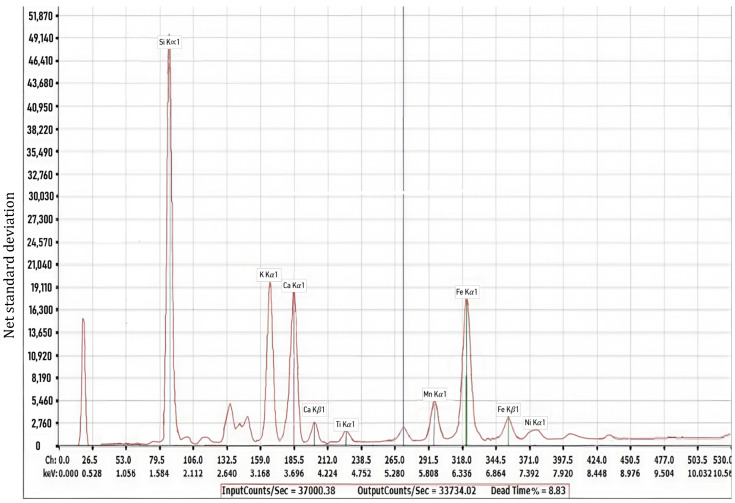
Components present in the wood ash.

The material samples were tested using XRD-XRF (40 kV, 10 mA). The results of the elemental analysis carried out on the wood ash are presented in Table 1 and Figure 3. The results showed that wood ash contained a range of elements with different net standard deviations. It was noted that the main element was calcium, as had been expected, with a net standard deviation of 2,585,050, followed by iron, manganese, and potassium, with net standard deviations of 210,743, 194,477, and 170,049, respectively. Minor elements present in the wood ash included sodium, zinc, and magnesium, with net standard deviations of 94, 771, and 780, respectively. The “net standard deviation” values presented in Table 1 reflect the statistical precision of the XRF measurements for each element. These values are not indicative of elemental concentration in terms of atomic or weight percent, but rather illustrate the reliability and strength of the detected signal. Higher net standard deviations correspond to more precise detection of the respective elements. Figure 3 shows the XRF analysis of the wood ash sample, displaying the various compounds present in the wood ash and their corresponding amounts in the y-axis. Once more, it can be seen that calcium is the major element, as shown by the sharp rise in the graph.

**Figure 4:**
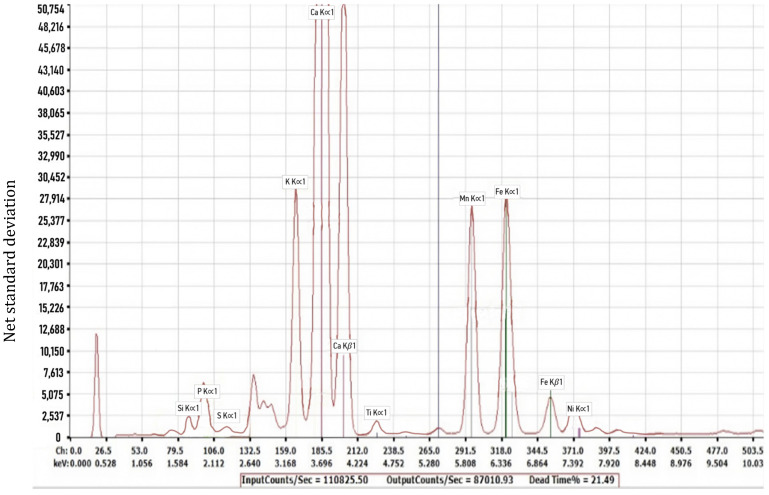
Components present in rice husk ash.

The results of the elemental analysis carried out on the rice husk ash are presented in Figure 4. It is observed that rice husk ash also contained a range of elements with different net standard deviations, with the main element being silicon, as had been expected, with a net standard deviation of 49,140. This can be seen from the sharp rise in silicon in the graph. Other elements present in the rice husk ash in substantial amounts included potassium, calcium, and iron. Minor elements present in the rice husk ash included sulphur and phosphorous.

Table 2 lists all observed elements in the ash whose mass concentration was above 0.07 wt%. The major elements were silicon, oxygen, and carbon.

The other elements that were reliably detected were the following (in order of decreasing concentration): Cl, Na, Zn, Rb, Zr, and Sr. When converting the elements to stoichiometric oxides, the major compound in the ash was SiO_2_, with approximately 87 wt%. The results of the XRD analysis are shown in the form of a diffraction pattern in Figure 4, which exhibits four reflections. The first weak reflection at 21.09° (2θ) and the third strong reflection at 26.63° matched perfectly with the 100 and 101 reflections of α-quartz (α-SiO_2_, space group P3121), respectively.

The small full width at half maximum (FWHM) of these two reflections (FWHM for the reflection at 26.63° was 0.04°) and their relatively low total integral intensity can be interpreted as originating from a few number of large grains of α-SiO_2_ that were preferentially orientated during the sample preparation for XRD analysis. These grains most probably stem from the soil and ground where the rice was grown and/or where the rice rusk was dried. The fourth weak and narrow reflection at 29.4° (interplanar spacing d = 0.303 nm) can be identified as belonging to calcite, which has its most intense 104 reflection located at a diffraction angle of 29.4°. This could also explain the small amount of calcium in the ash (0.4 wt%), which preferably exists in ambient conditions in the form of calcite. The second reflection at 21.52° was strong and very broad (FWHM = 6.8°, which is equivalent to X-ray apparent crystallite size of 0.7 nm, calculated using the Scherrer equation, Equation (4)):

(4) $$D=\frac{K\lambda }{\beta cos\theta }$$

where *D* is the crystallite size in nanometers (nm); *K* is the dimensionless shape factor, commonly taken as 0.9; *λ* is the wavelength of the X-rays (in nm or Ångströms); *β* is the FWHM of the diffraction peak in radians; and *θ* is the Bragg diffraction angle in degrees corresponding to the peak position.

Alternatively, the reflection at 21.52° can represent amorphous material, since amorphous phases produce broad humps instead of sharp peaks.

Taking into account the high concentration of SiO_2_ in the rice ash (Table 2), and matching this strong reflection with the XRD peak of amorphous SiO_2_ from treated rice straw ash [27] or amorphous silica (opal-A) [28], we can conclude that the major phase in the rice rusk ash was X-ray amorphous SiO_2_.

**Figure 5:**
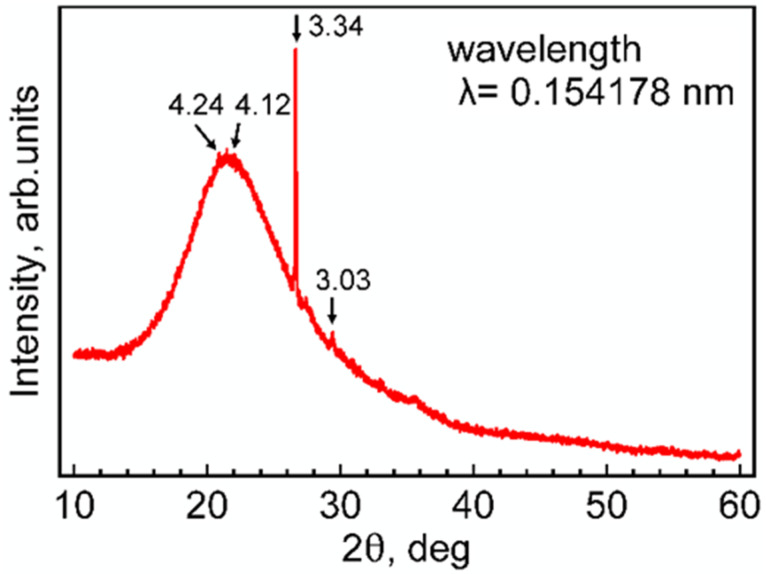
X-ray diffraction pattern of rice rusk ash.

The X-ray diffraction pattern of rice rusk ash is shown in Figure 5. The arrow points to the position of a peak, and the label is the interplanar spacing (d) calculated from Bragg’s law and expressed in angstroms.

### 3.2. Compressive Strength Tests

**Figure 6:**
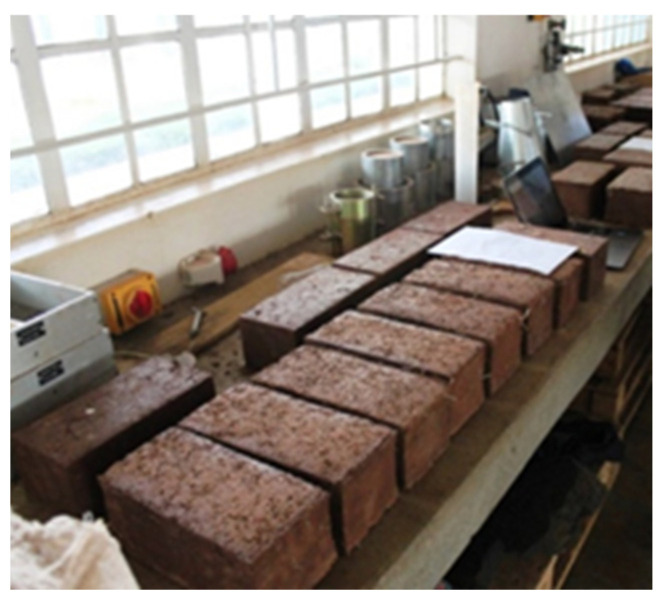
Test specimen used for compressive strength studies.

In the laboratory of Kenyatta University, test pieces were casted (Figure 6) and tested for their compressive strength. The results for compressive strength are shown in Table 3. It was observed that there was a general increase in compressive strength between batches number 1 and 3, with wood ash to rice husk ash ratios of 1:1 and 3:1, respectively.

Figure 6 shows blocks that were cast at the Civil Engineering workshops of Kenyatta University, in which soil was stabilized using rice husk ash. This gave good preliminary compressive strength results, as shown in Table 3. The compressive strength tests indicate that compressive strength directly depends on the lime-to-pozzolan ratio. The mechanism is discussed in the Discussion section.

### 3.3. Construction of Test House

**Figure 7:**
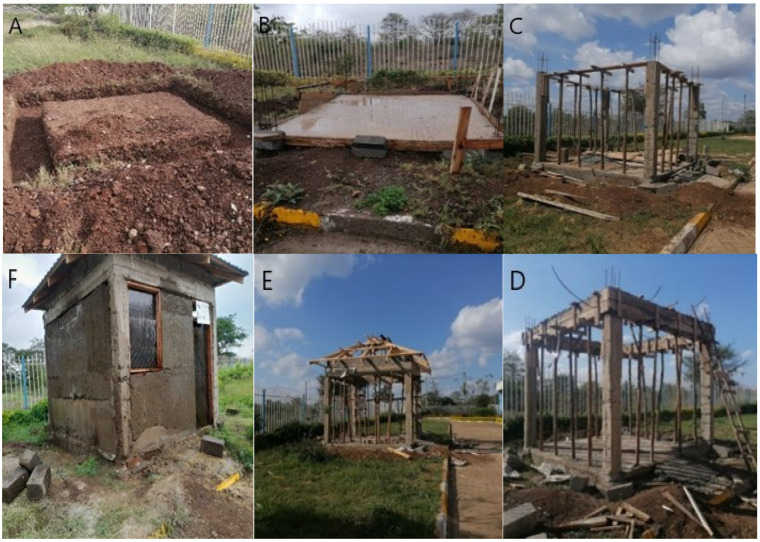
Test house construction in progress. Clockwise from top left: (**A**)—test house foundation; (**B**)—test house floor; (**C**)—support columns in place; (**D**)—ring beam in place; (**E**)—roof truss construction; (**F**)—test house in Kenyatta University.

The test house construction is shown in progress in Figure 7.

Due to the lack of access to a controlled environmental testing facility, such as a climatic chamber, it was decided to construct the test house in open outdoor conditions. This approach enables a real-world evaluation of the material’s long-term durability under natural climate stressors, such as solar radiation, humidity, rainfall, and temperature fluctuations typical for equatorial regions.

The house was built on the grounds of Kenyatta University, where it is exposed to ambient weather without additional protection or sheltering structures. The selected location allows for comprehensive observation of how the material responds to cycles of wetting and drying, biological factors such as mold and termites, and physical erosion.

The construction of the house was completed in 2022, and it is planned that after three full years of exposure, the structure will be dismantled in 2025. During dismantling, samples from different wall segments and internal layers will be taken and subjected to laboratory analysis. These tests will focus on mechanical strength retention, material phase composition (via XRD and FTIR), and structural integrity, including microcracking and carbonation depth.

This long-term exposure study is essential to validate the performance and reliability of the carbon-negative composite material under realistic operating conditions. In addition, the observed degradation or stability will guide the further development and potential scaling of this construction method. Despite limitations due to the absence of a climatic chamber, the open-air setup offers a robust and ecologically relevant framework for assessing the environmental sustainability and service life of novel bio-based construction composites.

## 4. Discussion

### 4.1. Efficiency of Apparatus

During the testing of the machine, it was shown that 0.7–1.0 m^3^ of mortar can be mixed in 30 min. The bottleneck was the cutting of the rice straw—the shredding of organic filler for the mortar will take several hours. Due to this, we decided to separate the cutting of the straw from the mixing of the mortar. When the straw has been prepared separately, the filling of the mixer with materials and water takes approximately 10 min. The mixing of the concrete takes another 10 min, and the timeframe for pumping concrete from the mixer to the formwork is similarly 10 min. Thus, 2 m^3^ of concrete can be mixed within an hour, which, considering the wall thickness of 40 cm and the wall height of 2.5 m, equals 2 m of built wall. Considering that the full length of the walls of a residential house with an area of 100 m^2^ is approximately 50 m, the construction of all walls takes about 25 h, or about three working days. The use of full-size equipment allows the construction work to be accelerated even further.

### 4.2. Effect of Ash Content on Compressive Strength

Regarding the wood ash to rice husk ash ratio (Table 3), it was also observed that there was a sharp decrease in compressive strength between batches number 3 and 4, with wood ash to rice husk ash ratios of 3:1 and 4:1, respectively. The compressive strength further decreases between batches number 4 and 5, with wood ash to rice husk ash ratios of 4:1 and 5:1, respectively. This clearly shows that the optimal mix ratio for maximum strength is the ratio 3:1, with a strength of 1.471 MPa. However, this strength was still low compared to the threshold of about 3 MPa of compressive strength required for blocks for use in wall construction.

According to building standards, for one- and two-storey homes, blocks with a minimum compressive strength of 2.9 MPa could be used. It is evident, therefore, that the mix of wood ash and rice husk ash alone was not able to provide adequate bonding for use in making a homogeneous and strong block/wall, and will require stabilization using either lime or a little cement to improve on its cementitious properties. This lack of bonding properties may have arisen because of the way the wood and rice husks were burnt to produce the ash. Controlled burning might be necessary if the mix is to gain bonding properties. When stabilizing the soil for construction, rice husk ash is usually combined with lime or any other cementitious material to improve the properties of the soil [29].

The analyzed data (Table 3) shows that with an increase in the amount of rice husk ash (RHA) in the blocks, the compressive strength of the block significantly decreases. The block shows the highest compressive strength at low quantities of RHA (5 wt% RHA). With an increase beyond 5 wt%, the compressive strength of the block goes below the minimum requirement for compressive strength.

This data would suggest that a further increase in RHA in the block would cause a greater reduction in the compressive strength of the block. Comprehensive tests are ongoing on different mixes of wood ash, lime, and rice husk ash to check its strength, durability, thermal insulation, and resistance to the adverse effects of climate, including resistance to water absorption. So far, the ongoing tests have shown some promising results. The first test house has been erected (see Figure 6). The ongoing work and tests will be reported in subsequent papers.

## 5. Conclusions

The development of environmentally sustainable and affordable construction methods is one of most vital problems for many regions of the world. The method developed by us is one solution. Rice straw is of low nutritional value as cattle feedstock, but both rice straw and rice husk, as well as sugarcane bagasse, contain a high amount of silica. Therefore, their ash can be used as an pozzolanic additive in the production of low environmental footprint concretes. Such materials can be used as a substitute for ordinary cement-based concrete in all regions of the world where rice and sugarcane are extensively cultivated. Using the appropriate equipment, a residential house for a family can be erected in 1–2 days and fully equipped in a few more days. The cost of the main construction (walls) is less than 1/10 of the cost of traditional construction. Previously, rice husk ash has also been used as concrete additive, but our technology allows for the use of cement to be avoided entirely. All of the material is based on waste, thereby preventing the enormous GHG emissions associated with the production of Portland cement. The material has been tested at the University of Tartu and Kenyatta University. During the project, a material mixing complex was designed at the University of Tartu and placed at Kenyatta University. In the current stage of the project, test houses are being designed and erected by the engineers and architects of Kenyatta University.

## Figures and Tables

**Table 1:** Analytical report for wood ash sample.

No.	Element	Line	Energy/keV	Net Standard Deviation
1	Na	K12	1.04	94
2	Mg	K12	1.254	780
3	Al	K12	1.486	3098
4	Si	K12	1.74	12,590
5	P	K12	2.01	16,718
6	S	K12	2.309	3889
7	K	K12	3.314	170,049
8	Ca	K12	3.692	2,585,050
9	Ti	K12	4.512	13,445
10	V	K12	4.953	3035
11	Mn	K12	5.9	194,477
12	Fe	K12	6.405	210,743
13	Ni	K12	7.48	31,591
14	Cu	K12	8.046	4080
15	Zn	K12	8.637	771

**Table 2:** Elemental and equivalent oxide composition of rice husk ash sample (expressed in weight percentages).

Element	wt%	Equivalent Oxide	wt%
O	50.6	─	─
Si	43.5	SiO_2_	86.8
C	2.5	CO_2_	8.5
K	0.8	K_2_O	0.9
Al	0.6	Al_2_O_3_	1.1
Fe	0.5	Fe_2_O_3_	0.6
Ca	0.4	CaO	0.6
P	0.3	P_2_O_5_	0.6
Mg	0.2	MgO	0.3
Mn	0.1	MnO_2_	0.1
Ti	0.1	TiO_2_	0.2
S	0.07	SO_3_	0.2

**Table 3:** Compressive strength of the blocks.

Batch Number	Wood Ash to Rice Husk Ash Ratio	Load at Failure (kN)	Maximum Compressive Strength (MPa)
1	1:1	233.811	5.759
2	2:1	189.985	4.679
3	3:1	143.863	3.543
4	4:1	98.782	2.433
5	5:1	80.651	1.986

## Data Availability

The original contributions presented in this study are included in the article. Further inquiries can be directed to the corresponding author.
